# Supplementary material for: Decoding technical multi-promoted ammonia synthesis catalysts
Source: Nat Commun. 2025 Aug 21;16:7820. doi: 10.1038/s41467-025-63061-6 (PMC12370944; doi:10.1038/s41467-025-63061-6)
Supplement: Supplementary file 1 — Supplementary Information [file 41467_2025_63061_MOESM1_ESM.pdf]

Supplementary Information

To

Decoding Technical Multi-Promoted Ammonia Synthesis Catalysts

Luis Sandoval-Díaz[1,*], Raoul Blume[2], Kassiogé Dembélé[1], Jan Folke[2], Maxime Boniface[1], Frank Girgsdies[1], Adnan Hammud[1], Zahra Gheisari[1], Danail Ivanov[1], René Eckert[3], Stephan Reitmeier[3], Andreas Reitzmann[3], Robert Schlögl[4], Beatriz Roldan Cuenya[4], Holger Ruland[2], Axel Knop-Gericke[2], Thomas Lunkenbein[1,*]

[1] Department of Inorganic Chemistry, Fritz-Haber-Institute of the Max-Planck-Society, Berlin, Germany

[2] Department of Heterogeneous Reactions, Max Planck Institute for Chemical Energy Conversion, Mülheim an der Ruhr, Germany

[3] Clariant Produkte (Deutschland) GmbH, Heufeld, Germany

[4] Department of Interface Science, Fritz-Haber-Institute of the Max-Planck-Society, Berlin, Germany

*to whom correspondence shall be addressed: lesandovaldi@fhi-berlin.mpg.de, lunkenbein@fhi-berlin.mpg.de

Table of Contents

Precatalyst characterization
Supplementary Fig. 1, 2 3
Activity tests at elevated pressures
Supplementary Fig. 3-5 4
Supplementary Note 1 5
Post-catalytic sample FIB lamella
Supplementary Fig. 6 6
Compilation of NAP-XPS data at varying temperatures
Supplementary Fig. 7, 8 7
Comparative semiquantitative activity data
Supplementary Table 1 8
Relevant XPS peak positions
Supplementary Table 2 9
Supplementary References 10

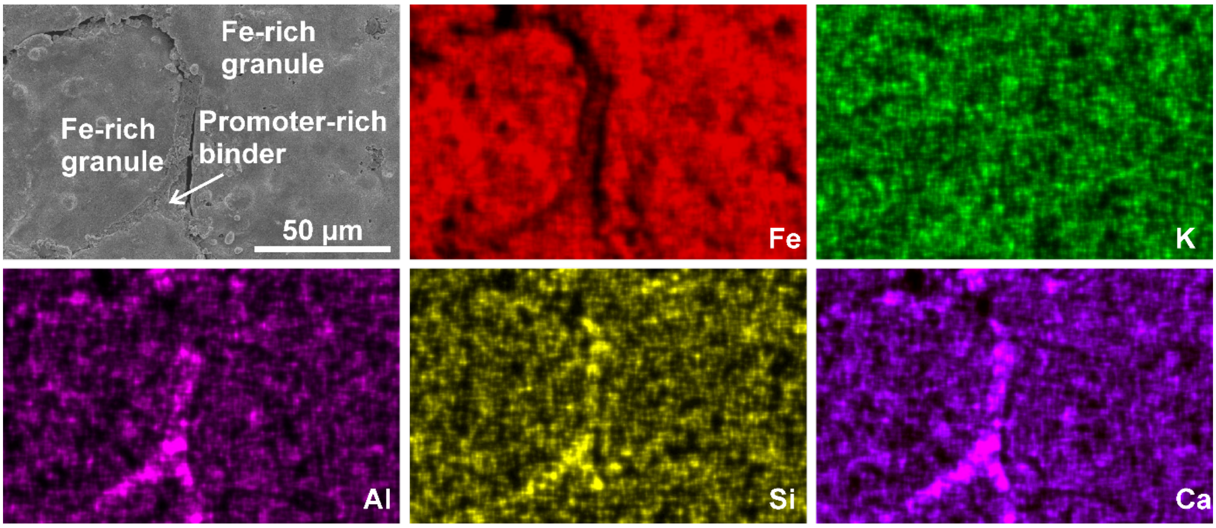

Supplementary Fig. 1. SEM-EDX elemental maps of technical, multi-promoted ammonia synthesis catalysts. The elemental distribution of Fe, K, Al, Si, and Ca of the passivated catalyst reveal Fe-rich regions held together by phases enriched with Al, Si and Ca.

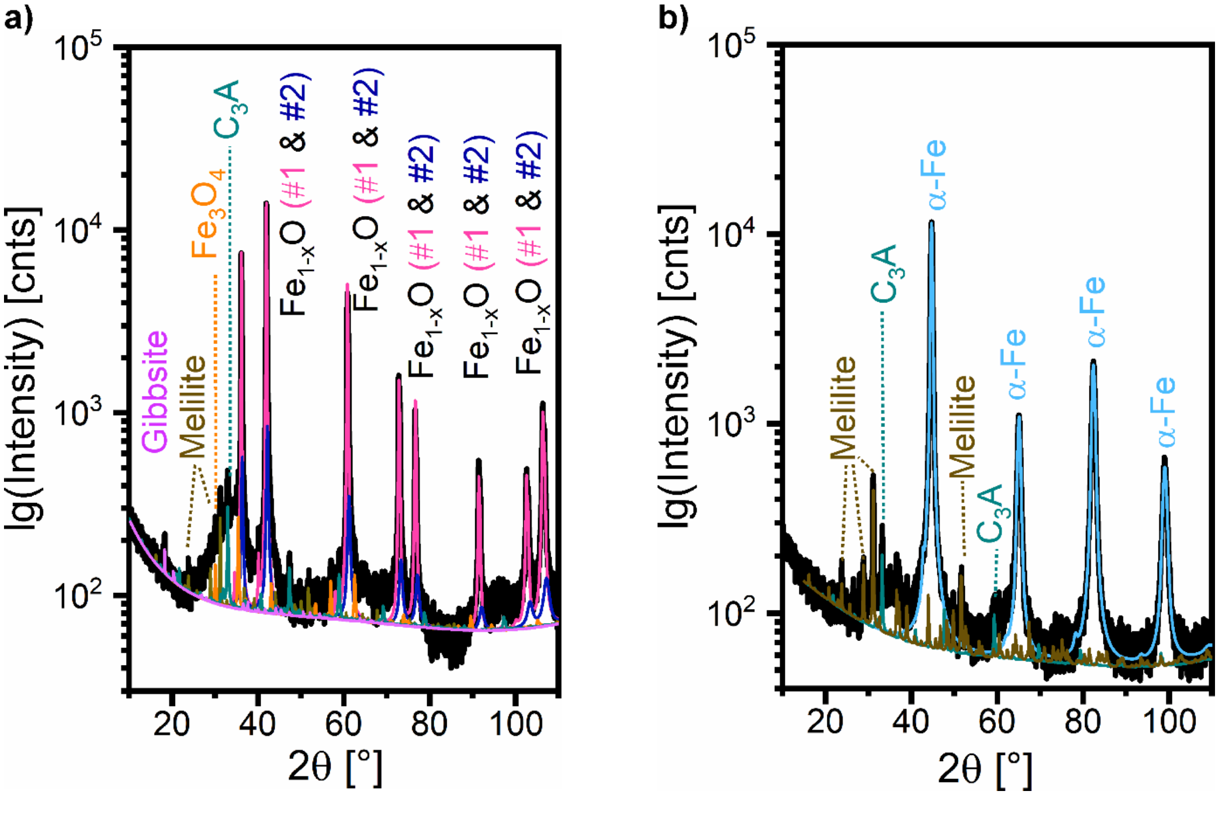

Supplementary Fig. 2. Powder X-ray diffraction patterns and crystalline phase assignment of technical multi-promoted ammonia synthesis catalyst a, before reaction and b, after 96 h of reaction at 90 bar. Gibbsite ($Al(OH)_3$), tricalcium aluminate (C_3A), magnetite, and a mixture of wüstite materials of slightly different compositions were detected. The wüstite denoted as #1 has the formula $Fe_{0.95}O$, and the #2 corresponds to $Fe_{0.92}O$. Melilite refers to the family of sorosilicate solid solutions of general formula $Ca_2(Al, Mg, Fe)$ $[(Al, Si)SiO_7]$, with end members gehlenite ($Ca_2Al[AlSiO_7]$) and akermanite ($Ca_2Mg[Si_2O_7]$) [1]. Additional characterization can be consulted in [2]

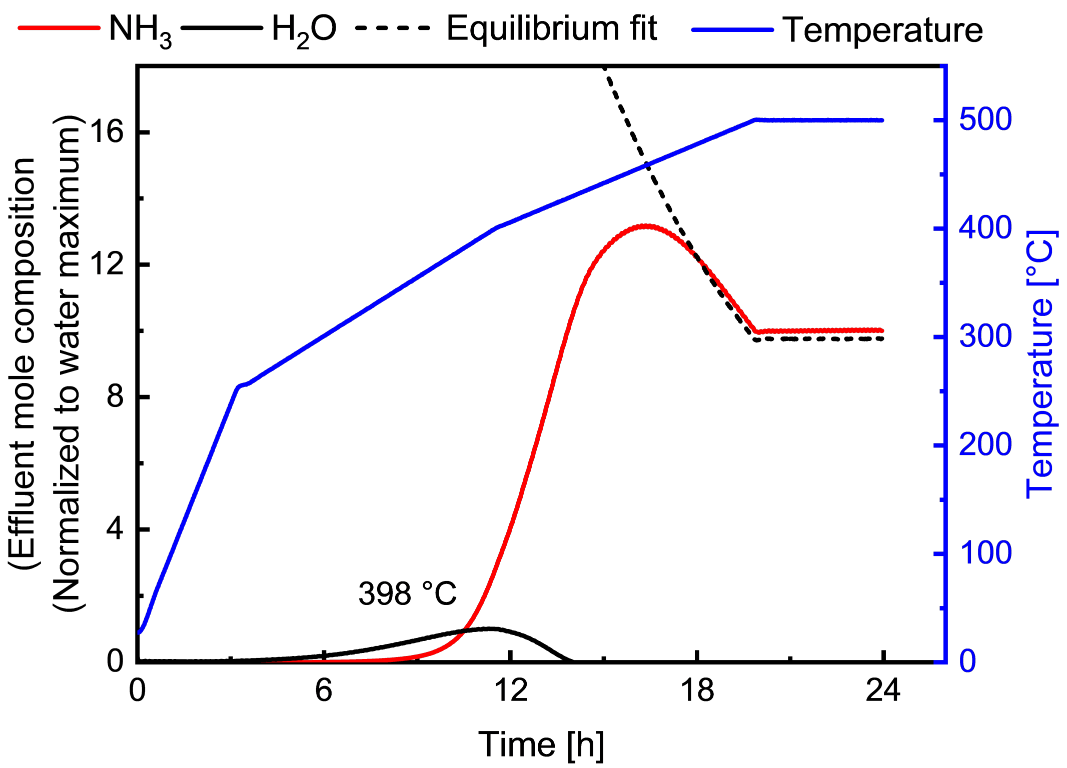

Supplementary Fig. 3. Ammonia production at 30 bar. The data shows the catalytic production of ammonia and water at 30 bar over the technical multi-promoted catalyst.

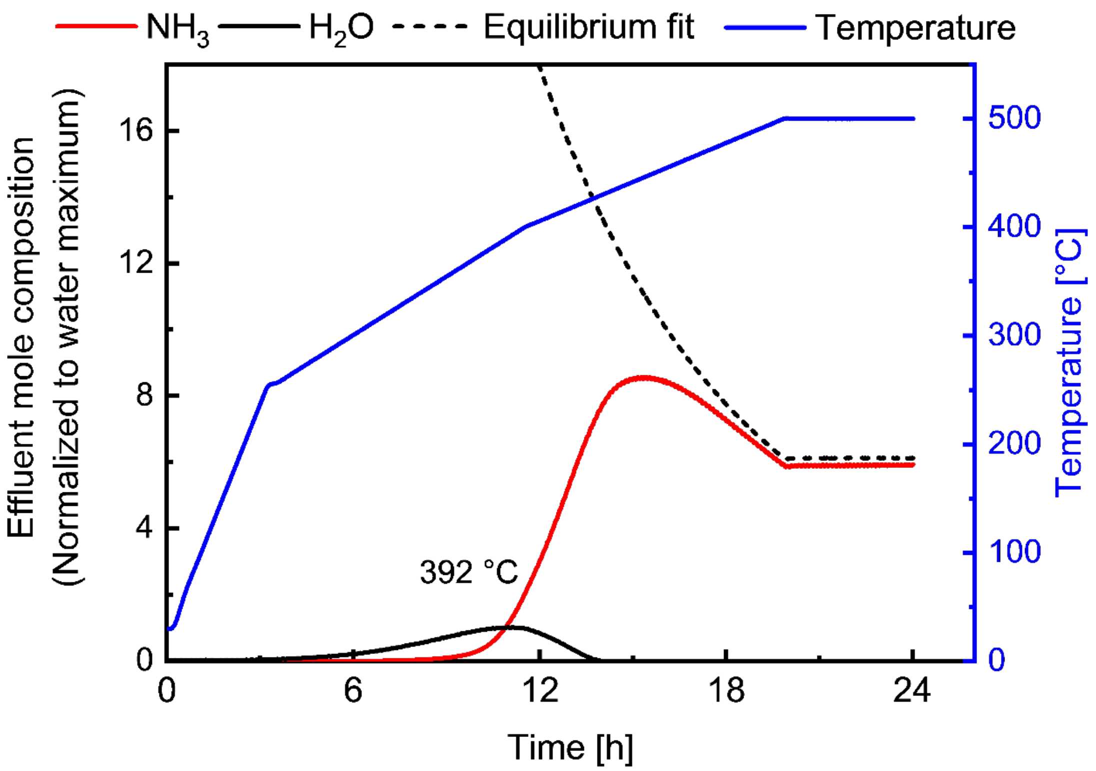

Supplementary Fig. 4. Ammonia formation at 15 bar. The data show the catalytic production of ammonia and water at 15 bar over the technical multi-promoted catalyst.

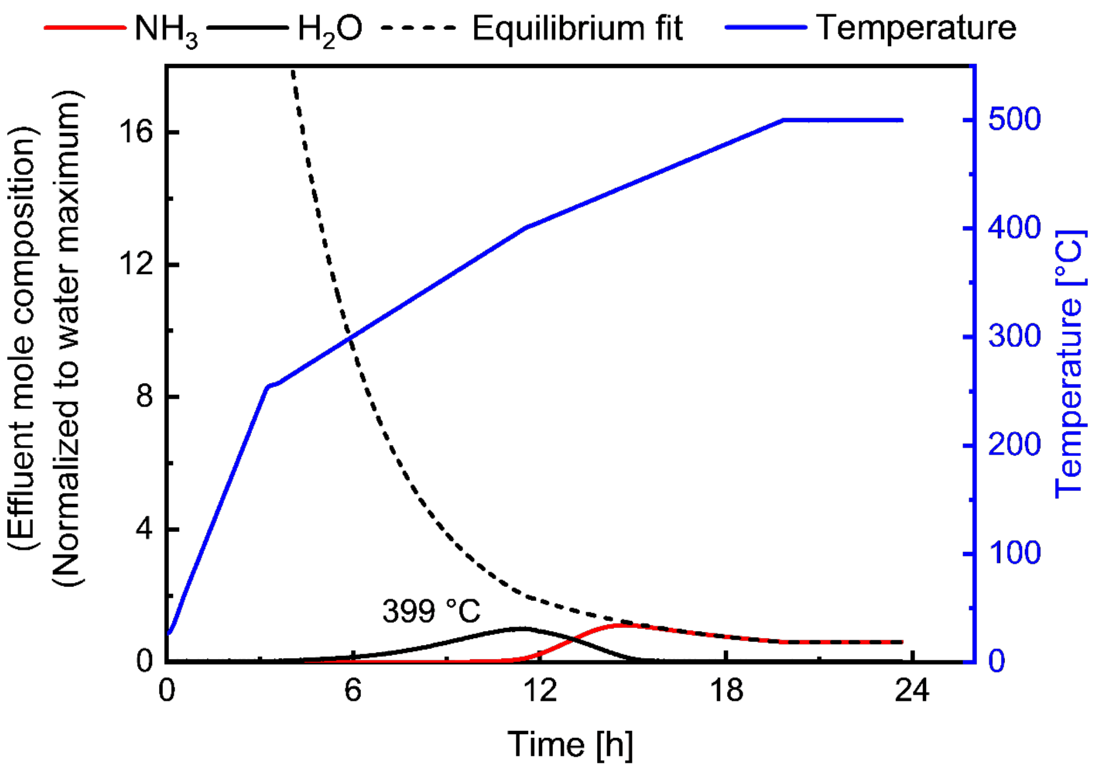

Supplementary Fig. 5. Ammonia formation at 1 bar. The data shows the catalytic production of ammonia and water at 1 bar over the technical multi-promoted catalyst.

Supplementary Note 1

The catalytic data presented in Supplementary Figures 3-5 illustrate the pressure dependance of the ammonia synthesis reaction. The catalytic production decreases with decreasing pressure due to thermodynamic constrains. For comparison, the effluent compositions were normalized to the peak of water. Figures S3-S5 show the same relevant features that were observed in the operando data of Figure 2 at sub-ambient pressure. These relevant features include a water peak in the interval between 210°C and 400°C, and the initiation of ammonia production simultaneously to the catalyst reduction.

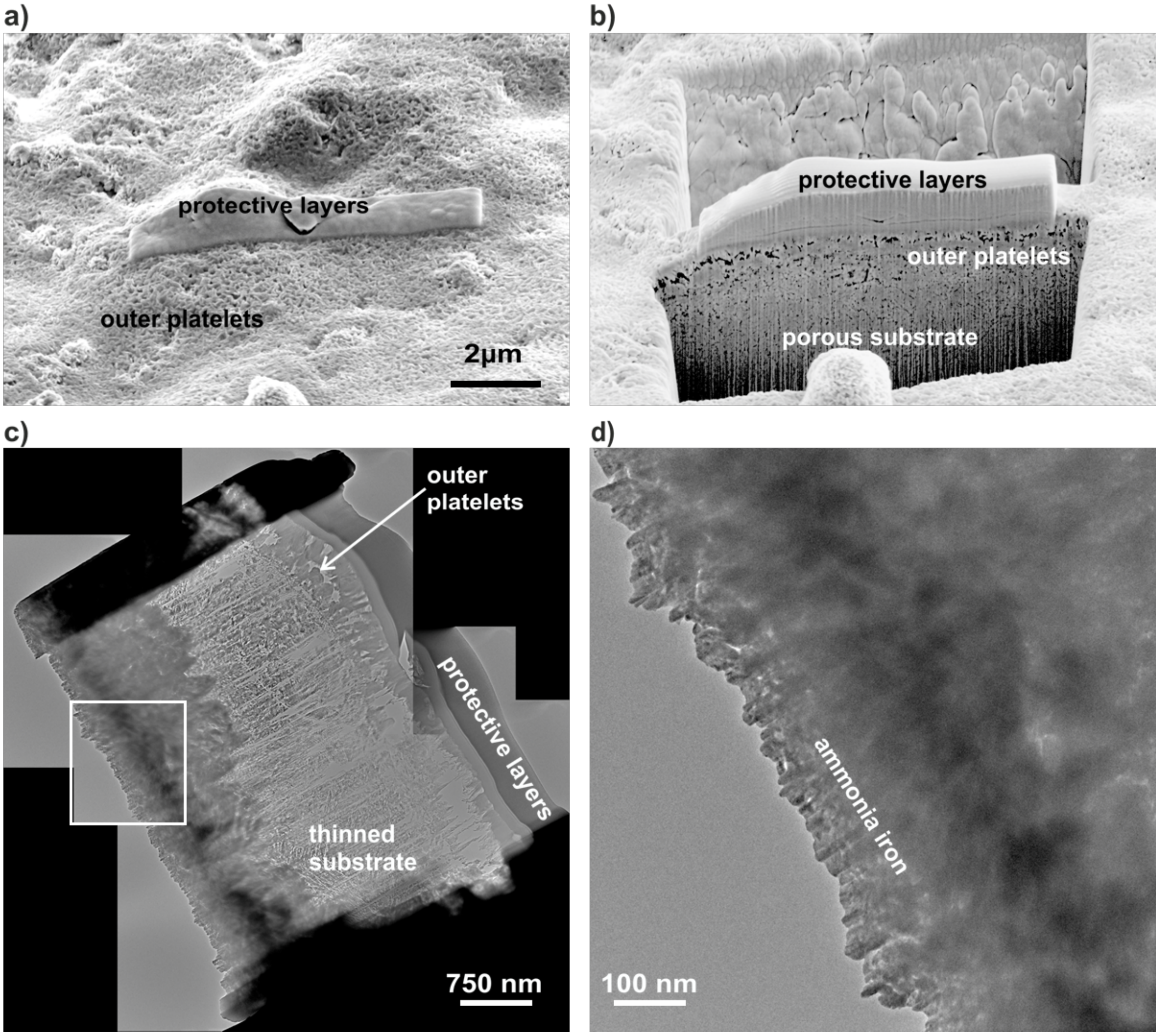

Supplementary Fig. 6. Additional electron microscopy characterization of technical, multi-promoted ammonia synthesis catalyst. a) and b). SEM images of the spent catalytic sample during the preparation and lift-out of a thin cross-section by Focused Ion Beam (FIB) milling. c) and d) BF-TEM overview images of the thinned cross-section showing the lamellar porous structure of the substrate giving rise to porosity.

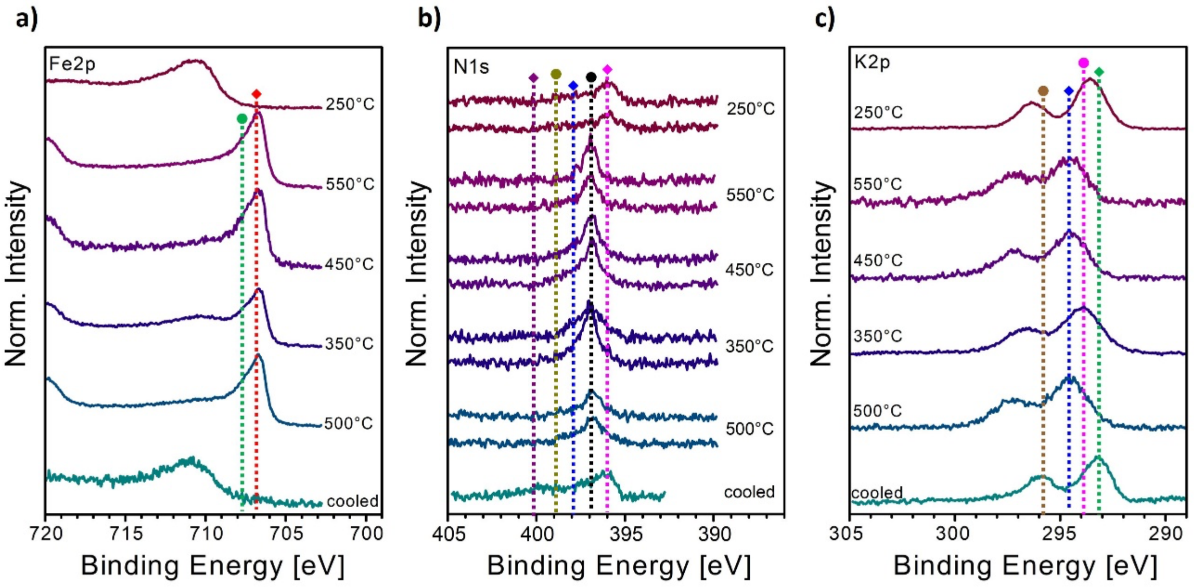

Supplementary Fig. 7. A cascade representation of the surface evolution of the catalyst. The panels showXPS data of a) the Fe2p$_{3/2}$ region, b) the N1s region, and c) the K2p region at different temperatures.

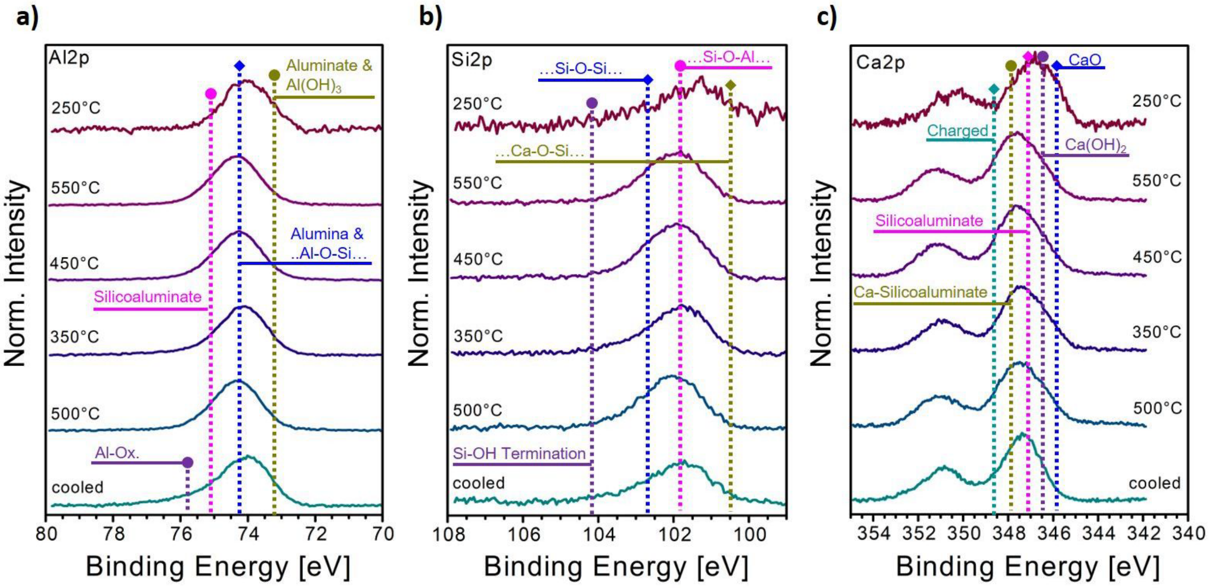

Supplementary Fig. 8. A cascade representation of the surface evolution of the catalyst. The panels show XPS data of a) the Al2p region, b) the Si2p region, and c) the Ca2p region at different temperatures.

Supplementary Table 1. Comparison between rate of ammonia formation in the OSEM experiment, iron single crystal catalysts and industrial ammonia conditions.

Catalyst type	$kg(NH_3)\ h^{-1}kg^{-1}$	$mmol\ (NH_3)\ h^{-1}g^{-1}$	$nmol\ (NH_3)\ s^{-1}cm^{-2}$
Multipromoted Fe[a]	4.34×10^{-4}	2.55×10^{-2}	0.47
Fe (111)[b]	154×10^{-4}	90.3×10^{-2}	~13.1
Fe (211)[b]	114×10^{-4}	66.9×10^{-2}	~9.7
Fe (100)[b]	23.5×10^{-4}	13.8×10^{-2}	~2.0
Fe (210)[b]	21.1×10^{-4}	12.4×10^{-2}	~1.8
Multipromoted Fe[c]	755	4.42×10^{6}	6.43×10^{5}

a. Measured in the OSEM experiment with a 1-point calibration of the QMS signal at m/z=15, 142.4mg of spent catalyst weight, and assuming a surface area of $15m^2g^{-1}$[3].

b. Values estimated from reference [4] at 20 bar and 450°C.

c. Values estimated from reference [5] at 100 bar and 450°C.

Supplementary Table 2. A compilation of the binding energies (BE) detected by NAP-XPS experiments and their assignments according to literature surveys.

Fe		References
**Binding energy (eV)**	**Species**	
706.9	Fe (0)-metallic	[6, 7]
707.7	Nitride, sulphide, hydride (Fe(II))	
708-714	Oxides (magnetite, wüstite, haematite)	[6, 7]
**N**		
**Binding energy (eV)**	**Species**	
395.9	Nitride (γ’)	
396.9	Atomic N	
397.9	Nitride (ε)	
399.1-400.2	Fe--NH_x	
**K**		
**Binding energy (eV)**	**Species**	
293.2	K_2O	[7, 9]
293.8	KOH	
294.8	K(0)-metallic, atomically dispersed K^+	
296.0	Charging artifact	
**Al**		
**Binding energy (eV)**	**Species**	
73.6	AlO_4-, Al-OH	[10-13]
74.3	Al_2O_3, Al-O, Al-O-Si	
75.2	Al_2O_3 (gamma), Al-O-Si + Ca	[10, 11, 14]
**Si**		
**Binding energy (eV)**	**Species**	
98.7	Si, Si/Fe	[10, 11, 15]
100.5	Ca-O-Si, SiO_x	[10, 11, 14, 15]
101.8	Si-O-Si, Si-O-Al, calcium silicoaluminate	[10, 11, 15]
102.7	SiO_2, Calcium silicoaluminate	[10-13, 15]
104.2	Si-OH termination	
**Ca**		
**Binding energy (eV)**	**Species**	
345.8	$Ca(OH)_2$	[10, 12-15]
346.5	CaO, CaS	[10, 12-15]
347.2	Calcium silicate, calcium silicoaluminate	[10-15]
347.9	hornblende	[10, 11, 15]
348.7	Charging artifact	

Supplementary References

[1] Chapter 12 - Phase Equilibria of Binary Systems, in: B. Fegley (Ed.) Practical Chemical Thermodynamics for Geoscientists, Academic Press, Boston, 2013, pp. 585-622.

[2] K. Dembélé, J. Wang, M. Boniface, J. Folke, L. Sandoval-Diaz, F. Girgsdies, A. Hammud, D. Kordus, G. Koch, Z. Gheisari, R. Blume, W. Jiang, A. Knop-Gericke, R. Eckert, S. Reitmeier, A. Reitzmann, R. Schlögl, B. Roldán-Cuenya, J. Timoshenko, H. Ruland, T. Lunkenbein, The Haber Bosch Catalyst from Solid state Chemistry to Mesotechnology, Advanced Energy Materials (2025) 2500159.

[3] J. Folke, K. Dembélé, F. Girgsdies, H. Song, R. Eckert, S. Reitmeier, A. Reitzmann, R. Schlögl, T. Lunkenbein, H. Ruland, Promoter Effect on the Reduction Behavior of Wuestite-based Catalysts for Ammonia Synthesis, Catalysis Today, 387 (2022) 12-22.

[4] G.A. Somorjai, N. Materer, Surface Structures in Ammonia Synthesis, Topics in Catalysis, 1 (1994) 215-231.

[5] M. Hattori, N. Okuyama, H. Kurosawa, M. Hara, Low-Temperature Ammonia Synthesis on Iron Catalyst with an Electron Donor, Journal of the American Chemical Society, 145 (2023) 7888-7897.

[6] Y. Qi, Q.-J. Li, Y. Wu, S.-j. Bao, C. Li, Y. Chen, G. Wang, M. Xu, A Fe3N/carbon composite electrocatalyst for effective polysulfides regulation in room-temperature Na-S batteries, Nature Communications, 12 (2021) 6347.

[7] Y. Joseph, G. Ketteler, C. Kuhrs, W. Ranke, W. Weiss, R. Schlögl, On the Preparation and Composition of Potassium Promoted Iron Oxide Model Catalyst Films, Physical Chemistry Chemical Physics, 3 (2001) 4141-4153.

[8] C.M. Goodwin, P. Lömker, D. Degerman, B. Davies, M. Shipilin, F. Garcia-Martinez, S. Koroidov, J. Katja Mathiesen, R. Rameshan, G.L.S. Rodrigues, C. Schlueter, P. Amann, A. Nilsson, Operando Probing of the Surface Chemistry during the Haber–Bosch Process, Nature, 625 (2024) 282-286.

[9] G. Franceschi, P. Kocán, A. Conti, S. Brandstetter, J. Balajka, I. Sokolović, M. Valtiner, F. Mittendorfer, M. Schmid, M. Setvín, U. Diebold, Resolving the Intrinsic Short-Range Ordering of K+ ions on Cleaved Muscovite Mica, Nature Communications, 14 (2023) 208.

[10] L. Black, A. Stumm, K. Garbev, P. Stemmermann, K.R. Hallam, G.C. Allen, X-ray Photoelectron Spectroscopy of the Cement Clinker Phases Tricalcium Silicate and β-Dicalcium Silicate, Cement and Concrete Research, 33 (2003) 1561-1565.

[11] C.D. Wagner, D.E. Passoja, H.F. Hillery, T.G. Kinisky, H.A. Six, W.T. Jansen, J.A. Taylor, Auger and Photoelectron Line Energy Relationships in Aluminum-Oxygen and Silicon-Oxygen Compounds, Journal of Vacuum Science & Technology, 21 (1982) 933-944.

[12] N.A.S. Webster, M.I. Pownceby, I.C. Madsen, A.J. Studer, J.R. Manuel, J.A. Kimpton, Fundamentals of Silico-Ferrite of Calcium and Aluminum (SFCA) and SFCA-I Iron Ore Sinter Bonding Phase Formation: Effects of CaO:SiO2 Ratio, Metallurgical and Materials Transactions B, 45 (2014) 2097-2105.

[13] N.A.S. Webster, M.I. Pownceby, J.R. Manuel, R. Pattel, J.A. Kimpton, Fundamentals of Silico-Ferrite of Calcium and Aluminum (SFCA) and SFCA-I Iron Ore Sinter Bonding Phase Formation: Effects of MgO on Phase Formation During Heating, JOM, 73 (2021) 299-305.

[14] F. Bellmann, T. Sowoidnich, H.M. Ludwig, D. Damidot, Analysis of the Surface of Tricalcium Silicate during the Induction Period by X-ray Photoelectron Spectroscopy, Cement and Concrete Research, 42 (2012) 1189-1198.

[15] L. Black, K. Garbev, P. Stemmermann, K.R. Hallam, G.C. Allen, Characterisation of Crystalline C-S-H phases by X-ray Photoelectron Spectroscopy, Cement and Concrete Research, 33 (2003) 899-911.
