# Supplementary material for: Decoding technical multi-promoted ammonia synthesis catalysts
Source: Nat Commun. 2025 Aug 21;16:7820. doi: 10.1038/s41467-025-63061-6 (PMC12370944; doi:10.1038/s41467-025-63061-6)
Supplement: Supplementary file 2 — Description of Additional Supplementary Files [file 41467_2025_63061_MOESM2_ESM.pdf]

Description of Additional Supplementary Files

File Name: Supplementary Movie 1
Description: The surface of the multi-promoted catalyst started to change during the reductive activation treatment. The drifting field of view of the OSEM images results from thermal expansion effects and chemical transformations.

File Name: Supplementary Movie 2
Description: A magnified and aligned ROI extracted from Supplementary Movie 1. Nanoparticle exsolution and surface texturization take place at this stage. The displayed images are representative for the temperature regime between 340°C and 450°C.

File Name: Supplementary Movie 3
Description: The surface of the multi-promoted catalyst continued to change during the reductive activation treatment at 500°C. Nanoparticle exsolution and growth gave rise to platelet-like aggregates at the catalyst surface. The displayed images are representative for TOS between 76.5h and 253h.

File Name: Supplementary Movie 4
Description: The segregated platelet material collapsed after continued exposure to the ammonia synthesis environment at 500°C. The remaining exsolved material transformed into crust-like and acicular morphologies. The displayed images are representative for TOS between 272h and 276.5h.

File Name: Supplementary Movie 5
Description: The material segregation was verified in a second run in the OSEM reactor from a fresh catalyst aliquot. The formation of platelets emerging from the porous substrate gave insight into the phase segregation during the ongoing activation. The displayed images are representative for TOS between 145h and 235h.
